# A Report of a Novel Mutation in Human Prostacyclin Receptor Gene in Patients Affected with Migraine

**Published:** 2017-07

**Authors:** Majid Kheirollahi, Mohammad Reza Pourreza, Fariborz Khorvash, Mohammad Kazemi, Gilda Amini

**Affiliations:** 1 Pediatric Inherited Diseases Research Center, Research Institute for Primordial Prevention of Non-Communicable Diseases, School of Medicine, Isfahan University of Medical Sciences, Isfahan, Iran; Department of Genetics and Molecular Biology, School of Medicine, Isfahan University of Medical Sciences, Isfahan, Iran.; 2 Department of Neurology, Isfahan University of Medical Sciences, Isfahan, Iran.

**Keywords:** *Migraine*, *Mutation*, *Prostacyclin Receptor Gene*

## Abstract

**Objective:** The human prostacyclin receptor gene encodes the human prostacyclin receptor, which is a part of the vasodilator system, during migraine attacks and almost certainly plays an important role in the mechanism of this disease.

**Method:** The present study aimed at determining any variants in PTGIR gene by means of PCR and direct sequencing. Blood samples were taken from the patients, and genomic DNA was extracted. Polymerase chain reaction was performed on the extracted DNA. The PCR products were then sequenced using the Sanger method.

**Results:** When reviewing the familial and clinicopathological history of the 2 patients, we found that both had symptoms of migraine with visual aura and that their mothers were also suffering from migraine. Their parents were not relatives prior to marriage. Direct sequencing of Exon 2 of the PTGIR gene showed the presence of 2 mutations. These mutations were heterozygous and made the following changes: g.1626T>A, c.754T>A, cDNA.867T>A, and p.S252T for the first mutation, and c.753C>T, cDNA866C>T, g.1625C>T, and p.C251C for the second mutation. The first mutation altered the amino acid and was a novel mutation. The second change was a conservative mutation that has already been reported.

**Conclusion:** The prediction results of silico studies indicated that the c.754T>A change would negatively affect the protein’s function and seemed to cause the disease. However, functional analysis is required to confirm the association between the variant and the disease.

Migraine, as the most common chronic neurological disorder, affects over 15% of the worldwide population. Migraine is a multifactorial disorder and is associated with environmental and genetic factors, but the underlying causes of migraine are unknown (1). Affected patients have moderate to severe pain. These headaches are often associated with autonomic nervous system symptoms and considered a neurovascular disorder (2). Some evidences suggest that the mechanism of disease begin in the brain, and then spreads to the blood vessels. Some researchers believe that the neural mechanisms have the major role, while others believe The PTGIR gene encodes a protein with 386 amino acids, which has a molecular weight between 37 and 41 kDa, depending on different states of glycosylation (8, 9). Binding of prostacyclin to hIP receptor activates this receptor and leads to activation of membrane-bound adenylyl cyclase. This process is followed by building the second messenger cyclic adenosine monophosphate (cAMP) and activation of different signals of cells (6). Since the first review on prostaglandins in migraine in 1985 (10), new information has been obtained on prostaglandins and migraine pathophysiology (11). The current theory about the migraine is that the deep structures of the brain are activated by unknown mechanisms. In addition, the trigeminal–vascular system (TVS) is activated by sensitization of peripheral and/or central pain pathways, and prostaglandins, as the local release of signalling molecules, play an important role (7). In addition, genetic diversity in the hIP receptor resulting in defect of the hIP receptor function is associated with increased disease severity in patients affected with migraine. In spite of important progress in our understanding of the structure and function of hIP receptor in general (12), the details remain largely unknown.

Because PTGIR is a part of the vasodilator system during the migraine attacks, it may have an important role in the mechanism of this disease. In the present study, PTGIR gene mutations in patients affected with migraine were considered and investigated.

## Materials and Methods

Blood sample was collected from the patients, and genomic DNA was extracted from 200 microliter peripheral blood of the 2 patients using PrimePrep Genomic DNA isolation kit (Genet Bio, Korea) according to the manufacturer protocol. Patients were referred by a neurologist after clinical evaluations. Specific primers (Table 1) for Exons 1 and 3 were designed for the intronic regions flanking the 3 exons of PTGIR using Primer3Plus website (http://www.bioinformatics.nl/cgibin/primer3plus/primer3plus.cgi) and according to the genomic sequence references available in the Genome Browser (http://www.ensemble.org).

Polymerase chain reaction was conducted on 50μL volume reaction under the following conditions: 1X buffer, 1.5 mmol/l magnesium chloride, 200 μmol/l dNTP, 400 nmol/L of each primer, 200 ng/μl DNA, and 2 U Taq DNA polymerase. The PCR was started for 4 minutes at 94°C and followed by 30 cycles of 30 seconds at 94°C, 30 seconds at 60°C, and 60 seconds at 72°C, and was ended by 5 minutes at 72°C. Prior to sequencing, the PCR products were stained with ethidium bromide and visualized on a UV transilluminator following 1.5% gel electrophoresis. 

The PCR products were then sequenced using a cycle sequencing kit on an automated DNA sequencing machine (BigDye Terminator v3.1 and 3730XL DNA analyzer, Applied Biosystems). All sequences were matched to the PTGIR reference sequence using NCBI blast, and in silico analysis was performed to determine the effect of the variants.

The study was conducted according to the Declaration of Helsinki and approved by the Ethics Committee of Isfahan University of Medical Sciences. Informed consent was obtained from the patients.

## Results

***Case Report***

When reviewing the familial and clinicopathological history of the 2 patients, we found that both patients had a migraine with visual aura and their mothers were also suffering from migraine. Their parents were not relatives prior to marriage. 

***Sequencing and In Silico Analysis***

**Figure 1:**
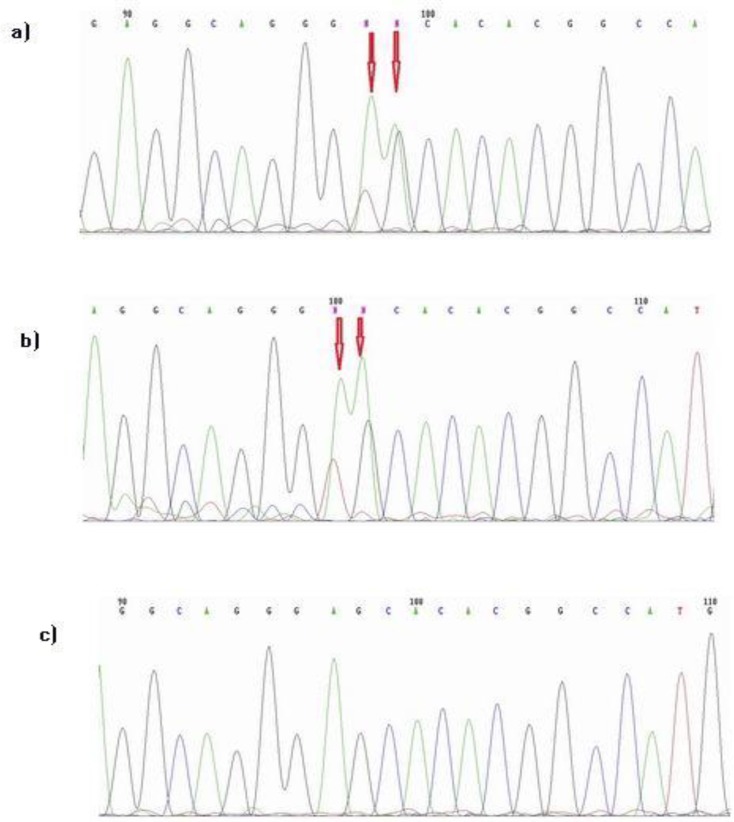
Direct sequencing analysis of exon 2 of the human prostacyclin receptor gene. a and b) The presence of mutations in two patients. The first mutation was heterozygote that made the following changes: a threonine instead of a serine at position 252 (e.g., p.S252T), and the second change was c.753C>T, cDNA866C>T and g.1625C>T. c) the normal sample (without mutation).

Direct sequencing analysis of the Exon 2 of the PTGIR gene showed the presence of 2 new mutations in the 2 patients. The first mutation was heterozygote that made the following changes: a threonine instead of a serine at position 252 (e.g., p.S252T), and the second change was c.753C>T, cDNA866C>T and g.1625C>T. However, this change in nucleotide could not cause the substitution of amino acid (p.C251C) (Figures 1-a and 1-b). Normal sequence is presented for comparison in Figure 1-c.

The first mutation altered the amino acid and was a novel mutation, and the second change was a conservative mutation that has already been reported. I-Mutant, SNPs3D, and MutationTaster 2.0 were applied to understand the effect of the variant on the protein structure, function, and its association with the disease. I-Mutant 2.0 assesses the effect of the variant on the protein stability based on the sign of the free energy change value (sign DDG). SNPs3D evaluates the functional impact of the variant based on alignment or structure. MutationTaster 2.0 measures functional consequences of the amino acid substitution. The prediction results predicted that the variant would negatively affect the protein’s function and may cause the disease (Table 2). However, functional analysis is required to confirm the association between the variant and the disease.

## Discussion

Prostacyclin (PGI2), which is released from vascular endothelial cells, acts as a potent vasodilator. In addition, PGI2 inhibits aggregation of platelet (antithrombotic) and moderates proliferation–migration–differentiation of vascular smooth muscle cells (antiatherosclerotic).

Seven transmembrane-spanning G-proteins, which are the recipients of PGI2, coupled the receptor (GPCR), known as the human prostacyclin receptor or hIP.

Numerous SNPs in the PTGIR gene encoding the hIP receptor has been revealed by recent genomic sequencing (13-16).

**Table1:** Primer sets that were used for amplification of human prostacyclin receptor Exons

**Name**	**Sequence**		**Product size(bp)**
PTGIR-F1 PTGIR-R1	GGGGCAGAGAGAGGAAATGA AATCCGCCATCCCAGGTC		972
PTGIR-F2 PTGIR-R2	AGGGACATCTGAGTGGGCT CCCACGATGTCTCACCTCTT		967
PTGIR-F3 PTGIR-R3	ATCCTGCTGGCCCTCATGA GGACCAAGCCTCTGTCTGAT		838

**Table2:** In Silico Analysis of the Variant (c.754T>A). I-Mutant, SNPs3D, and Mutation Taster 2.0 were applied to understand the effect of the variant.

Software	I-Mutant 2.0	SNPs3D	Mutation Taster 2.0
Prediction	Decrease stability DDG = -0.38	Deleterious SVM = -2.68	Disease causing

Comparison of the available data of this mutation with that of previously biochemically characterized mutants has revealed a correlation between genetic variants in the hIP receptor that cause defects in hIP receptor function, with the severity of disease in patients with coronary artery disease (17, 18). Moreover, advances in the discovery of naturally occurring receptor changes have helped facilitate further structure-based predictions and pinpoint dysfunctional receptor focal points (e.g., nonsynonymous mutations resulting in amino acid substitutions or deletions). Understanding the structure and function of human prostacyclin receptor at the molecular level will not only help better develop hIP-specific pharmaceutical agents, purification methods, functional assays, and antibodies, but will also help develop insight on principles common to G-protein coupled receptors and other prostanoids in general. 

## Limitations

Our limitation in this study was patient collaboration.

## Conclusion

In conclusion, biochemical analysis of genetic variances in functional assays can support the discovery of functional correlations of naturally occurring genetic variation and disease. In addition, information about the structural requirements of the receptor for the function can help guide rational drug design and development of novel therapies for the disease.
